# Evaluation of a community-based integrated care model (CIE) for frail older people in rural Foshan, China: study protocol for a stepped-wedge cluster randomized controlled trial {1}

**DOI:** 10.1186/s13063-023-07328-7

**Published:** 2023-05-08

**Authors:** Fengjiao Xie, Shuang Wen, Aiwen Deng, Jianhao Chen, Ribo Xiong

**Affiliations:** 1 Department of General Surgery, The Third Affiliated Hospital of Southern Medical University, Guangzhou, China; 2 Department of Thoracic Surgery, Nanfang Hospital, Southern Medical University, Guangzhou, China; 3 Department of Rehabilitation, The Seventh Affiliated Hospital, Southern Medical University, Foshan, China

**Keywords:** Integrated care, Chronic care model, Community-based eldercare, Community-based rehabilitation, Primary healthcare, Stepped-wedge trial, Frail elderly, Quality of life, Process evaluation

## Abstract

**Background:**

While community-based eldercare has proven to be effective in qualitative studies, there is limited evidence on the effectiveness of this geriatric care model in rural communities where caring for older people is traditionally the responsibility of family members, but a formal long-term care was recently introduced in China. CIE is a rural community-embedded intervention using multidisciplinary team, to provide evidenced-based integrated care services for frail older people including social care services and allied primary healthcare and community-based rehabilitation services.

**Methods:**

CIE is a prospective stepped-wedge cluster randomized trial conducted at 5 community eldercare centers in rural China. The multifaceted CIE intervention, guided by chronic care model and integrated care model, consists of five components: comprehensive geriatric assessment, individualized care planning, community-based rehabilitation, interdisciplinary case management, and care coordination. The intervention is rolled out in a staggered manner in these clusters of centers at an interval of 1 month. The primary outcomes include functional status, quality of life, and social support. Process evaluation will also be conducted. Generalized linear mixed model is employed for binary outcomes.

**Discussion:**

This study is expected to provide important new evidence on clinical effectiveness and implementation process of an integrated care model for frail older people. The CIE model is also unique as the first registered trial implementing a community-based eldercare model using multidisciplinary team to promote individualized social care services integrated with primary healthcare and community-based rehabilitation services for frail older people in rural China, where formal long-term care was recently introduced.

**Trial registration {2a}:**

China Clinical Trials Register (http://www.chictr.org.cn/historyversionpub.aspx?regno=ChiCTR2200060326). May 28th, 2022.

## Introduction {6a}

Reforming eldercare delivery to meet the multiple needs of an increasing number of frail population is a top policy agenda in many countries around the world [1]. The challenges are particularly acute in rural China, where informal care network is weakened due to the out-migration of young working-age adults to urban areas and decreasing birth rate [2]. The issue is further compounded by the fact that government-run institutions and senior care homes in China are reserved for those with no children, no income, and no known relatives [3]. Privately operated facilities are seen as the fastest way to meet the demand. However, the initiatives have apparently not served their intended purpose partly because of its inconsistency with filial piety of traditional Chinese Confucian philosophy and the stigma of nursing homes. Specifically, the current practice of private-for-profit services caused a huge financial burden for older residents and their families, with additional challenges coming from the lack of professionals and paraprofessionals [3]. Provision of community-based care for frail older people through integrating existing human resources with suitable local conditions is a potential strategy to deal with the diverse needs of older people [4]. Under this model, older people do not need to move out of the home and the community provides day care in the form of visiting service or staying in a daycare center [4]. Yet, implementation of such an innovative eldercare model is challenging.

The integration of health services and social services for senior residents has gained tremendous attention in recent years [5]. Evidence suggests that older people will have better health outcomes, enhanced satisfaction and decreased costs if a person-centered coordinated care is provided in the community [6, 7]. Though widely acknowledged and pursued, several gaps in implementing such models exist. First, in China, routine healthcare is practiced through a tiered healthcare delivery system whereby each level of healthcare facility (tertiary, secondary, and primary) would deliver care according to their designated functions [8]. This adds to the list of barriers to prevention and treatment of cases and scale up of services in eldercare institutions because they are financed, governed, and managed separately. Primary healthcare providers are in a central position to coordinate the changing needs of the ageing population in many countries, yet their role of coordinating with specialty care has not reached its full potential in China [9]. Second, many eldercare practice modes only provide several simple medical examinations and treatments, such as health checkup, prescribing drugs, and basic first aid [4]. Such activities may be effective in yielding positive changes in certain groups, but, in the real world, a model with a more person-directed approach to engaging individuals in decision-making is needed. In contrast to the geriatric model, several integrated care approaches at the community setting have been proposed, but they have only targeted a single chronic disease (e.g., stroke), outcomes (e.g., quality of care, costs), or process (communication, team building) [10–12].

Further evidence is also necessary on an integrated care model for frail older adults. An important distinction between chronic disease and frailty is that frailty is more often associated with functional impairments and physical inactivity that require a restorative or enabling approach [13]. This study aims to address this gap. We developed an integrated care model guided by the concepts and practice of community-based rehabilitation (CBR) for frail older people, named Community-based Integrated Eldercare (CIE). Unlike a typical Western model of CBR that is integrated into primary healthcare systems, CBR service in primary healthcare centers of China remains in a low quality due to poor education and systematic training on general practitioners even though these centers have been equipped with the newest equipment and technology [14]. This current CIE intervention includes the elements of a CBR delivered by specialists in physical and rehabilitation medicine (based on a more level model of delivery of rehabilitation services). In addition, CBR is a specific intervention appropriate for the Chinese culture using traditional methods (e.g., moxibustion, traditional Chinese medicine fumigation, massage, cupping therapy and skin scraping) as the practical entry point to promote inclusion and participation of people in the local community. Another unique aspect of this study is the proposed CIE model will be tested at community eldercare centers (CECs) in rural China, a novelty formal long-term care form developed in a few cities, but was recently expanded to the rural population [4].

The objective of this study is to evaluate the effectiveness of a community-based, multidisciplinary, integrated care model for the frail older people named Community-based Integrated Eldercare (CIE). The specific aims are:

1. Evaluate the efficacy of CIE model to improve functionality and dependency, cognitive and emotional status, and quality of life and enable frail older people to live in the community for longer.
2. Process evaluations to gain insights into applying the same model to different settings in the future.

## Methods

### Study design {8}

**Fig. 1:**
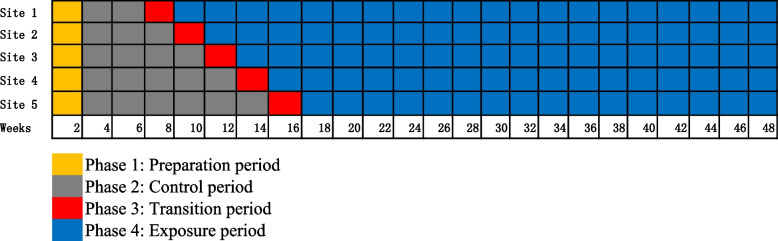
Stepped-wedge study design in 5 community eldercare centers (clusters)

This study will adopt a multicenter, prospective, unidirectional randomized controlled trial using a stepped-wedge cluster design with repeated cross-sectional samples. Four phases will be sequentially rolled out over 48 weeks: preparation, control phase (standard care exposure), transition phase (intervention introduction), and intervention exposure (Fig. 1).

The stepped-wedge design offer advantages in pragmatic community trials when an intervention can only be delivered in a staggered manner to account for practical logistics constraints. Additionally, stepwise implementation allows all participants to receive the intervention at some time during the study which is ethically appropriate [15].

### Study setting {9}

The model is implemented in Lishui county, located in the middle of Pearl River Delta in Southern China. At the end of 2019, the population of Lishui county was 580,000, 22.3% of which are above 60 years of age [16]. The county is divided into 2 blocks with a total of 36 villages. A majority of Lishui’s rural young population have migrated to urban areas for better opportunities and income. As a result, older people is left behind and forced to take care of themselves. In terms of eldercare and healthcare services, Lishui has 18 Community Eldercare Centers (CEC), 1 Primary Healthcare Center (PHC), and 18 Primary Healthcare Stations (PHS) staffed by 130 general practitioners (GPs) and 79 nurses, along with a tertiary comprehensive hospital which provides additional human resources (such as geriatrician and rehabilitation specialist) for geriatric care. Community-based care for older people is primarily delivered by social workers at the CECs and the GPs also visit older people on a 3-monthly basis. The tertiary hospital has in-patient and out-patient services for older adults with multiple health needs, and limited follow-up care following discharge.

### Study sites and participants {10}

Five CECs were purposively selected based on geographic location and representativeness of the frailty in Lishui county.

Study sites were purposively selected in collaboration with villagers committee and Bureau of Civil Affairs according to the following criteria: (1) sites where CBR services were delivered by the tertiary hospital; (2) at least 20,000 visits per year altogether in selected CECs. Based on previous data in the county [17], we assumed each site may be able to see on average 40 frail older people and they are present at least 100 days per year (eight per month); (3) routine eldercare available onsite; (4) no previous introduction of interventions/organizations for integration or increasing services, apart from CIE that is considered as standard-of-care throughout the whole study period.

Study participants consists of all the adults aged 65 or older who are present for care in the selected CECs and agree to participate during the study period. Inclusion criteria are as follows: (1) with household registration in Lishui county; (2) not planning to move away from Lishui county in the prospective follow-up of the study; (3) Chinese version of Mini-Mental State Examination score ≥ 20; (4) written informed consent to participate in the study. For those who had difficulty in writing, informed consent was obtained from their spouses/children. The oral informed consent was recorded when the older people or their family members cannot write. Exclusion criteria are as follows: (1) on a waiting list for a nursing home; (2) with a terminal medical condition; (3) older adults who have psychiatric disorders or other illnesses that require hospitalization; (4) older adults who are currently receiving specialist geriatrician intervention and/or care coordination.

### Intervention: the CIE model {11a}

#### Theoretical rationale

The CIE model is guided by the evidence-based Chronic Care Model (CCM) and the multidisciplinary integrated care model through a restorative or enabling approach specified for the community-based eldercare system in China [18, 19]. Minkman et al. theorized how to implement integrated care and identified two most fundamental principles: engaging and empowering people and communities, population-oriented, and focusing on promoting health [20]. Given the fact that frailty is more often associated with functional impairments and physical inactivity that require a restorative or enabling approach beyond the scope of a traditional CCM [13], we constructed four key components of the CIE model as follows: comprehensive geriatric assessment, individualized care planning, community-based rehabilitation, interdisciplinary case management, and care coordination. The CIE model was originally developed by our research team, which has expertise in chronic disease management and eldercare system in rural China. The model was further refined based on literature and expert review, consultations with representatives from villagers committee, feedback from field staff, and also a pre-test of intervention components. Details of the CIE model are listed in Table 1.

**Table 1:** Description of the CIE model: components and implementation information {13}

Components	Outcomes	Providers	Place	Time	Dose
Comprehensive geriatric assessment (CGA)	Profile registration form including medical history, functional scales and service demand evaluation in social work practice	GP-rehabilitation doctor-social worker	At each CEC or at each home	At T0	At least Once Anytime needed (condition change of residents)
Individualized care planning (CP)	A written checklist form	GP-rehabilitation doctor-social worker Residents/family members/caregivers	At each CEC or at each home	Right after the CGA is done at T0	At least Once Anytime needed (condition change of residents)
Community-based rehabilitation (CBR)	Individualized, written plans with goals, timeline, and therapies	Specialists in physical and rehabilitation medicine	At each CEC or at each home	When CGA and CP are done	At least twice a week
Interdisciplinary case management (ICM)	A written checklist form	GP-rehabilitation doctor-social worker Residents/family members/caregivers Facilitated by enablement officer and coordinator team	At each CEC	When a relevant case is found	At least once a month
Care coordination (CC)	Shared information among stakeholders Coordinated action among service providers	The onsite coordinator team facilitated by enablement officer	-	The whole study period	-

#### Components of CIE

1. Comprehensive geriatric assessment (CGA): It is a multidimensional, multidisciplinary diagnostic process that aims to determine a frail elderly person’s medical, psychosocial, and functional capacities and problems [21]. Unlike traditional geriatric care models, care teams in CIE consist of a general practitioner, a nurse, a rehabilitation doctor, and a social worker. We adopted certain domains identified and recommended by the WHO as needing assessment [21]. Based on these domains, different tools have been embedded in the profiling. Thus, by completing CGA, members in the care team obtain a list of valuable and valid data for each resident. These results work as a decision-support tool to develop an overall care plan and long-term follow-up.
2. *Individualized care planning(CP)*: CP provides a guide for organizing and prioritizing care delivery. Based on information from CGA results, the care team in each CEC develops a care plan for each individual specifically addressing how individual or family preferences are or can be incorporated in care planning processes or the care plan itself. To support CP, the CIE program provides a set of checklist forms that involve actions of each party. Unlike the traditional, provider-driven process of data collection, assessment, and care plan development, the checklists are based on the key principles of a person-directed care planning: autonomy, personhood, and the strengths-based approach, viewing each resident as a unique individual who brings distinct and critical perspectives to care planning [22]. Care teams can also revise the checklists to ensure care plans remain aligned with the needs and preferences of individuals. This practice of integrating the person’s goals in care planning and iteratively revising care plans is rare in eldercare facilities in China.
3. *Community-based rehabilitation(CBR)*: Unlike traditional studies applying CBR that target a single disease, CIE targets frail older people with multiple needs, for which CBR is essential for achieving and maintaining optimal functioning. The key activities of a CBR program comprise of organizing training sessions, providing assistance (health education, assistive devices, and housing adaptation), and extending social and recreational support [23]. CBR is not a new concept, but almost all the eldercare facilities in rural China admitted they did not do CBR at all due to limited resources, or CBR was delivered by volunteers or GPs who have limited systematic training in rehabilitation [23]. In the CIE model, specialists in physical and rehabilitation medicine are responsible for CBR. Guidelines for the CBR were developed based on formal research studies, diverse experiences of disability, best practices drawn from similar approaches, as well as the research team’s own expertise [24].
4. *Interdisciplinary case management (ICM)*: Case management is a collaborative, client-driven process characterized by exchanging ideas and opinions among team members for the provision of quality health and support services [25]. Through ICM, care teams have a shared vision on the content of the care, enabling consensus among stakeholders and reducing duplication of services. For frail older adults, they do not have to repeat their stories to every member of the care team. In the CIE model, a geriatric nurse practitioner is assigned as a case manager who supports the multidisciplinary team by arranging meetings and streamlining the necessary exchange of information. During the meeting, care plan will be approved, actions and care paths will be discussed, and agreements will be made about the care to be deployed and the activities of all persons involved.
5. *Care coordination (CC)*: CC is a key construct for delivering integrated care for community-dwelling residents [26]. The CIE model facilitates communication between service providers and frail older residents in the community using evidence-based reports, reflecting a novel eldercare mode in rural China. The CIE model provides individualistic reports to four involved stakeholders: CECs administrators, contracted eldercare service providers (rehabilitation professionals and social workers, primary health care staff), and frail older residents and their family members/caregivers. Administrators receive a summary report regarding residents profile, care needs, and care plans. Moreover, the report for the elderly and their caregivers is summarized in a relatively simple way. Explicit attention is paid to the necessary support and guidance of the caregivers. The research team and care team also uses WeChat, a free mobile messaging application for communication throughout the program implementation and evaluation.

#### Procedure

The intervention procedures are as follows: The CIE model uses an enablement officer from the research team who is an advanced health professional specializing in geriatric care and understands the philosophy and specific details of the model. The enablement officer facilitates the implementation of the model in each community-based eldercare centers by arranging a time to meet representatives of all participating providers to train and empower them. A coordinator team consisting of a GP from primary health care institution and a community staff is then set up in each center and coordinates participants’ community care by linking them with existing services. ^**{26a}**^The coordinator team contacts eligible individuals and discuss the project with them. Upon completion of their verbal consent, a multidisciplinary team involving a GP, a rehabilitation doctor, and a social worker conducts CGAs and develops care plans based on a set of guidelines. The proposed plans are then reviewed, modified, and confirmed by older residents and their family. Explicit attention is paid to the necessary support and guidance of the caregivers. The responsibilities and activities of the involved professionals are formalized in agreed plans. Thus, the coordinator team functions as an entry point through which older adults can access the expertise and services of all health and social care professionals and organizations. The coordinator team also supports the multidisciplinary team by arranging meetings and streamlining the necessary exchange of information. ^**{11b}**^A secondary line geriatric specialist is invited to join the interdisciplinary discussion if the needs are multiple or of a complex nature. A trial steering group in each community-based eldercare center consisting of two administrative staff from the center and the villagers’ committee is responsible for running the trial day-to-day and providing organizational support. They will meet leaders of the coordinator team and the multidisciplinary team once a month. There are no restrictions regarding concomitant care during the trial.

### Comparator {6b}

During the control period, usual care will be administered by staff from each CEC and primary healthcare station. Services provided by social workers are formalized in agreed protocols with the county government. Healthcare services provided by GPs are identical to the usual practices. While social workers generally do some assessments, neither CGA nor CBR tends to be conducted by the multidisciplinary team or in a systematic way. Consultations with the older residents and their caregivers during the implementation process are very limited. The execution of care delivery is left to the discretion of care staff of each CEC and primary healthcare station, following their existing practice patterns. Restrictions regarding concomitant care during the trial are not available either.

### Patient and public involvement

There were public and patient involvements in the design of the protocol.

### Outcomes {12}

The outcomes will be measured four times at (1) T0 (2 weeks, the end of preparation period), T1 (6 weeks; the end of observation period), T2 (28 weeks; 20 weeks after the beginning of the intervention), and T3 (48 weeks; the end of the intervention). Outcome data will be collected by external research assistants trained by the CIE research team (Table 1).

#### Primary outcomes

- *Frailty*: The Chinese version of Groningen Frailty Indicator (GFI) will be used to measure frailty with a cut-off point of 3. It is composed of 15 items and divided into four domains. The total score ranged from 0 to 15 with each item having a score of 0 or 1. The GFI demonstrates good reliability with Cronbach’s alpha coefficient of 0.64 and acceptable construct and criterion validity as described by Tian et al. [27]. 
- *Quality of life*: The Chinese version of the 36-item Short Form Health Survey (SF-36) is administered which is comparable to the original scale with respect to reliability, convergent, and discriminant validity tests. It generates scores across eight dimensions of health that can be summarized into Physical Components Summary (PCS) and Mental Components Summary (MCS). The SF-36 has proved useful in monitoring population health outcomes in clinical practice, and evaluating treatment effects [28].
- *Mental well-being*: Depressive symptoms will be measured using the Center for Epidemiological Studies Depression Scale (CES-D). The CES-D consists of 16 negative affect and 4 positive affect items. Each item is accompanied by a standard 4-point Likert scale of potential responses. Higher scores on the CES-D indicate more depressive symptoms. The Chinese version of this scale has been validated and extensively used in community-based population [29]. Seven-item scale for General Anxiety Disorder (GAD-7) will serve as the self-rated measure of anxiety. The GAD-7 score is calculated by assigning scores of 0, 1, 2, and 3 to seven questions respectively. Scores of 5, 10, and 15 were taken as the cut-off points for “mild,” “moderate,” and “severe” anxiety, respectively. This instrument showed good reliability and validity in Chinese population [30].
- *Social support*: Likert scale is composed of three subscales that can be classified into informational support, emotional support, and household activity support. Its reliability and validity have been confirmed in a previous study [31].

#### Secondary outcomes

- *Patient-reported measurement of the shared decision-making process*: Patient experience of shared decision-making process will be measured using the CollaboRATE tool. The tool will assess the extent to which each of three core shared decision-making tasks (or dimensions) are present in a clinical encounter: (1) explanation of the health issue, (2) elicitation of patient preferences, and (3) integration of patient preferences. Each question will be scored on a 5-point Likert scale, with responses of 0 (no effort was made), 1 (a little effort was made), 2 (some effort was made), 3 (a lot of effort was made), and 4 (every effort was made) [32].  
- *Collaborative function of an interprofessional team*: The level of collaborative practice among interprofessional team will be measured by The Collaborative Practice Assessment Tool (CPAT). The CPAT survey includes 56 items across nine domains identifying strengths and weaknesses in their collaborative practice. CPAT is a valid and reliable tool for measuring healthcare team members’ perceptions of working collaboratively [33].

### Sample size {14}

The sample size for this stepped-wedge controlled randomized trial (SW-CRT) is calculated to detect minimal significant effects on the variable of quality of life: accepting an alpha risk of 0.05 and a beta risk of 0.20 in a bilateral contrast, referring the study of Ana.et al. [34]. Assuming the intracluster correlation coefficient (ICC) to be 0.01 based on Boorsma et al.’s study [35] and correlation coefficient to be 0.25 based on the ratio between the ICC and the correlation coefficient used in the study of Muntinga et al. [36], a minimum cluster size of 76 individuals is required to detect the expected intervention effect with 80% power at the 5% significance level.

Based on our earlier survey study on community-dwelling older people and available data on the characteristics of long-term care residents [17], we expect a 20% loss to follow-up due to mortality, impossibility, or unwillingness to participate further, 16 more individuals would be required, the resultant sample size is determinate in 92 individuals per cluster per period.

### Participant recruitment {15}

Prior to the recruitment procedure, we hosted a meeting with heads of villages, administrative staff from community-based eldercare centers, and representatives from social work organizations on October, 2020. We first explained the aims and target population of this eldercare model to each attendee. Then the healthcare program was introduced including adopted therapies, frequency of rehabilitation services, and follow-up visits. After addressing their questions, they were encouraged to spread the information. Brochures regarding this eldercare model were also given to them. The coordinator team, enrolled in the study, will select eligible participants from the roster that includes residents over 65 years old provided by the county government in each CEC. We used roster as it is the most complete, comprehensive, and accessible pool of older residents in Lishui county. Information sessions organized by research team will be held at a convenient time in each CEC for service providers. The coordinator team will then contact eligible individuals to discuss the CIE intervention and study requirements, inviting them to provide informed consent for inclusion in the study.

Participation in this study is voluntary. Eligible residents will be reassured that the decision not to be involved in the study would not have any impact on their current or future access to geriatric or primary healthcare services. There is no anticipated harm and compensation for trial participation. For residents who participated in this study, post-trial care is not available.

### Randomization and blinding {16a,16b, 16c, 17a}

Community eldercare centers (CECs, clusters) are recruited, and then they are allocated to different steps using computer-generated random numbers. The random-sequence remains concealed from all stakeholders during the first 6 weeks of the study so that all sites start usual care blinded of when the CIE model will start. Then an independent biostatistician discloses to the principal investigator which five sites have been randomized to start the CIE model in the first step. Each site is simply informed 2 weeks prior to each site starting to recruit residents. The five sites undergo a transition phase of 2 weeks with training and coordination before crossing to the intervention phase. All other sites continue usual care, still blinded on which time the CIE model will be offered, until the last 2 weeks before the next step. We do not anticipate any requirement for unblinding, but if required, the enablement officer, coordinator team, multidisciplinary team, and advisory committee will have access to group allocations and any unblinding will be reported.

Since blinding is not possible for trial participants and care providers in such a stepped-wedge design where all participants receive interventions sequentially, several procedures are used to minimize contamination and bias. First, clusters are geographically dispersed. Second, data collection staff is separate from the intervention team and blind to the on-going intervention study. Data analysts will not be aware of the identification information on participating CECs and individuals.

### Data collection, management, and monitoring {18a, 18b, 19}

Trained external assessors are responsible for the collection and maintenance of study records and data using web-based data collection and storage programs along with mobile tools such as an iPad and/or cell phone.

At the study center in Lishui, data will be entered electronically into a database. ^**{27}**^All information of the participants will be kept confidential using an identification code for each participant. During the electronic recording, a designated data entry person is trained for data plausibility to avoid erroneous data entry. Data will be entered within 1 month after each measurement point. After freezing the data, any further changes to the database will be impossible. For quality assurance, all entered data will be shared with a biostatistician via a password-protected file storage server by the principle investigator on a monthly basis. The biostatistician will collate the data, review data quality in terms of numbers, consistency, and completeness^**{21b,29}**^. A data monitoring committee was not considered as this was a low-risk intervention.

An advisory committee consisting of one principal investigator, one enablement officer, and three experts (medical ethics, social science, and public health) randomly selected from think tank by the county government will be formed to monitor the implementation and research. The committee checks with the onsite coordinator team on the status of implementation of the model at least every 3 months. A short-form report is filled out to be submitted to the research team^{5d,11c,21a,23}^.

### Statistical analysis {20a, 20b, 20c}

Data analysis will be performed using STATA 15. Baseline differences between clusters will be tested using Pearson’s chi-square statistic and ANOVA.

Statistical analysis is based on the Hussey and Hughes model for the analysis of cross-sectional SW-RCT designs [37]. Generalized linear mixed models (GLMM) will be used to determine the effect of the CIE model on the primary outcomes. Two random effects will be introduced, one at the cluster (eldercare centers) level and the other at the individual (frail older people) level. In addition to assessing the intervention effect, we will also investigate whether the time of intervention impacts the effectiveness of the intervention by adding an interaction term between time and intervention as a fixed effect. A secondary analysis will be conducted to adjust for baseline covariates to account for potential confounding effects. The same GLMM will be used to model the primary outcomes. Estimates of difference and 95% CIs will be calculated.

Qualitative data from interviews or focus groups will be analyzed using content analysis, document analysis, etc.

The analysis is based on intention to treat (ITT) principle. Multivariable normal models will be used for imputation of missing values to enable ITT analysis. A *P* value < 0.05 will be considered statistically significant.

### Adverse event reporting {22}

Any adverse events, including exercise injuries, will be reported in detail by the study assistant of the research group using the adverse event case report form.

### Dissemination {31a}

The results of this study will be disclosed in peer-reviewed journals. The main findings of the study will also be shared with all participants and disseminated to researchers and health service providers.

Any changes to this protocol will first be reported to the county government. Then the principle investigator will notify the coordinator team and a copy of the revised protocol will be sent to the principle investigator to add to the original file. Any deviations from this protocol will be fully documented using a breach report form. We will also update the protocol in the clinical trial registry.

## Discussion

This study is designed to evaluate whether CIE improves geriatric care for community-dwelling frail people against the backdrop of rapid demographic shifts in China. As far as we know, this is the first registered trial of community-based eldercare model for frail residents promoting integrated care services in rural China. We have adopted a holistic response to frailty that blends a traditional chronic care model with a restorative or enabling approach through CGA and multidimensional interventions to encourage frail older people to resume activity and regain independence. It also focuses on aligning the work of existing care professionals and convincing them to work together from a patient-centered viewpoint rather than fragmented care. CIE model is innovative in by embedding CBR in eldercare through specialists in physical and rehabilitation medicine rather than GPs and related organizational structural changes to eldercare delivery. Finally, CIE model is innovative in its reliance on community-based eldercare, a novelty eldercare model which has been developed in a few developed cities in China, but is recently introduced in rural communities [4].

The effectiveness and dissemination of the community-based integrated care model depend on its successful implementation. Research has suggested that a better understanding of the context of best practices in integrated care is needed to distinguish between generic and context-specific barriers to and facilitators of implementation [20]. Several activities have been deployed to ensure that these challenges are overcome. First, before the study starts, multiple stakeholders, such as administrators, service providers, and elderly representatives, are installed in a steering committee. The steering committee forms coordinator teams and multidisciplinary teams which promote multifaceted CIE intervention and incorporate the preferences and choices of the frail older people and their families. Second, in the policy level, the county government issued the decision on strengthening eldercare services and put forward the goal of coordinating social care services with other primary healthcare services, such as allied health and rehabilitation services. The infusion development of healthcare and eldercare is laid down in the formalization of agreements on the regional policy. The project is also supported financially by health insurance system which is universal and mandatory. Certain interventions such as CBR and social care services that are not yet included in the benefit package are covered by reimbursement agreement, conditional on assessment results. Third, the project is eventually secured by the strong willingness of older residents to accept new responsibilities thrust upon them [17].

The feasibility of the experiment will also be enhanced by a SW-RCT design which has several strengths for this study. Roll-out of the project to all participating CECs at different time-points during the intervention period is practical to implement and well-suited to the evaluation of health service delivery interventions. This design allows all clusters eventually receive the intervention which may alleviate ethical and/or community concerns.

There are several anticipated challenges. Secular trends unrelated to the intervention exposure, time-varying intervention effect, and treatment effect heterogeneity make results subject to contamination by confounding variables. These risks will be taken into consideration in the statistical analysis plan including adjustments with baseline covariates. It is conceivable that constrains in financial resources may decrease the fidelity of participating CECs to the implementation of the CIE program. Irrespective of the design chosen, the main concern of this study is the differences found in the experimental group might be the result of the additional attention given by both care professionals and external assessors rather than an integrated care. However, it may be difficult to disentangle the relative impact of the different aspects of the intervention. And the increased attention for the frail elderly is also one of the goals of the CIE model.

Demonstrating the benefits of integrated care for frail older people will provide strong supportive evidence to catalyze the widespread implementation of this intervention. The integration of clinical big data and web/mobile phone-based platform will be the best preparation for the implementation of the geriatric care in the future. Future research will also include a comprehensive quality management index system for evaluating CIE, using the Delphi method to provide direct feedback to professionals and administrators, that can be applied in a large-scale effectiveness study.

## Trial status

The current protocol is version 3 of 10–12-2021^**{3}**^.

China Clinical Trials Register (http://www.chictr.org.cn/historyversionpub.aspx?regno=ChiCTR2200060326).

Recruiting will start in October 2022 and patient recruitment will be completed around November 2022.

## Data Availability

The datasets used and/or analyzed during the current study are available from the corresponding author on reasonable request.
